# Supplementary material for: Conserved regulatory logic at accessible and inaccessible chromatin during the acute inflammatory response in mammals
Source: Nat Commun. 2021 Jan 25;12:567. doi: 10.1038/s41467-020-20765-1 (PMC7835376; doi:10.1038/s41467-020-20765-1)
Supplement: Supplementary file 1 — Supplementary Information [file 41467_2020_20765_MOESM1_ESM.pdf]

Supplementary Information

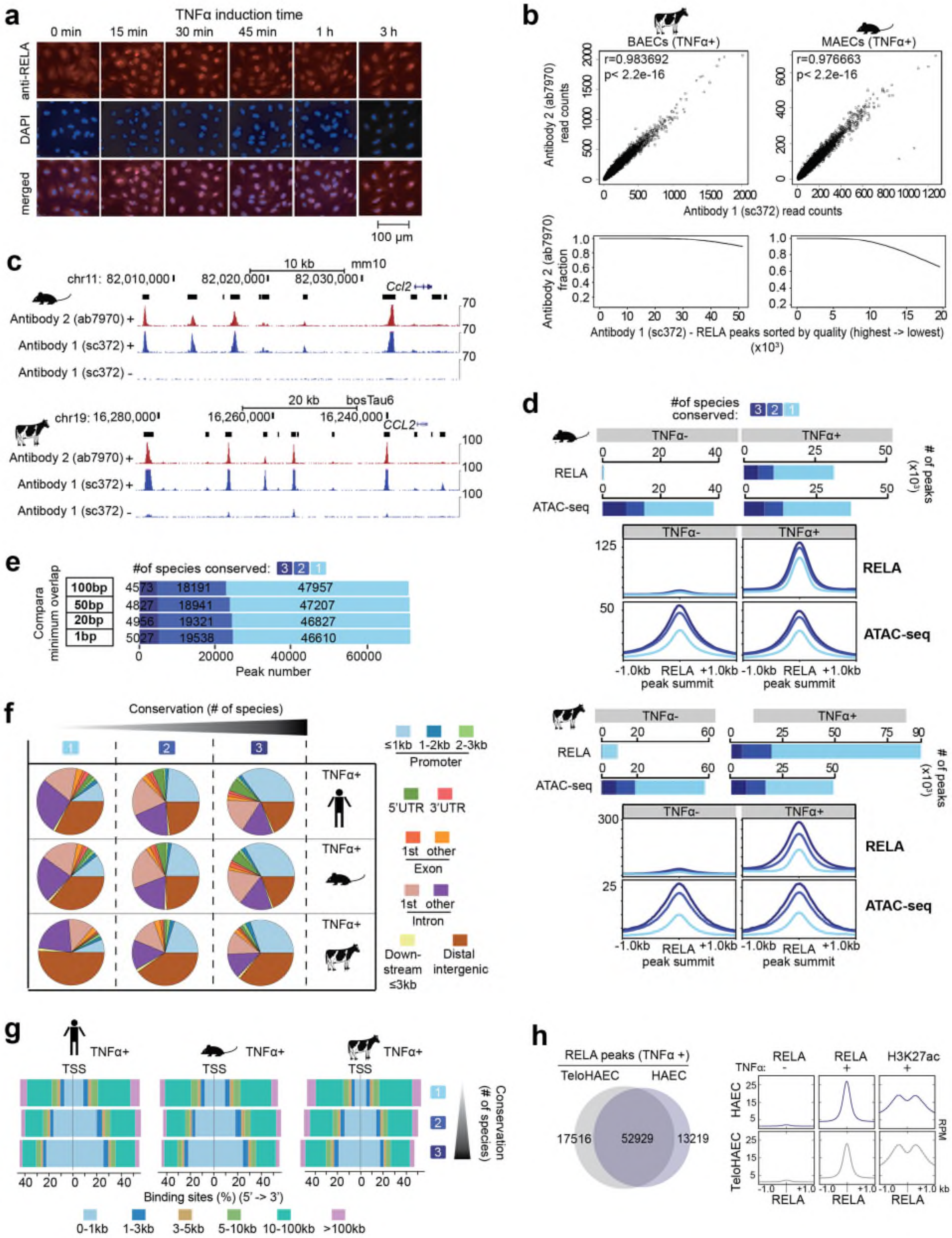

Supplementary Figure 1. Quality control, validation and features of RELA ChIP-seq peaks across species.

a, Representative immunofluorescence images showing translocation of RELA (Cy3 - red) into the nucleus (DAPI - blue) of human aortic ECs (HAECs) in a TNFα induction time-course (10 ng/mL). The experiment was repeated twice independently with similar results. **b,** Dot plots (top) showing correlations of read counts at RELA peaks identified using the two RELA antibodies (sc372 and ab7970) in TNFα-stimulated mouse aortic ECs (MAECs) and bovine aortic ECs (BAECs). Pearson correlation coefficient was used to measure the strength and significance of the correlation. Plot (bottom) indicating ranked sc372 RELA peaks (MACS2 FDR q-value) plotted against the fraction of overlapping ab7970 RELA peaks at each rank position. **c,** The *Ccl2*/*CCL2* locus showing representative RELA ChIP-seq signals (Reads per Million: RPM) derived from the two RELA antibodies (sc372 and ab7970) in TNFα-stimulated (45-min, 10 ng/mL) MAECs and BAECs. Black bars indicate RELA peaks. **d,** Stacked bar charts (top) showing the number of 3-species conserved (dark blue), 2-species conserved (blue), and species-specific (light blue) RELA ChIP-seq and ATAC-seq peaks in TNFα-stimulated (45-min, 10 ng/mL) MAECs (top) and BAECs (bottom). Profile plots (below) indicating ChIP-seq and ATAC-seq signals in RPM with mapped reads centered on the RELA peak summits. The plot lines indicate mean RPM ± SEM. **e,** Using 1, 20, 50, and 100bp RELA peak overlaps in the ENSEMBL-EPO multiple sequence alignment as the criteria for conserved orthologous RELA binding in HAECs. **f,** Pie charts showing genomic annotations of the 3-species conserved, 2-species conserved and species-specific RELA peaks in TNFα-stimulated (45-min, 10 ng/mL) HAECs (UCSC hg19 knownGene database), MAECs (UCSC mm10 knownGene database) and BAECs (UCSC bosTau6 ensGene database). **g,** Stacked bar charts depicting the distance and orientation of the 3-species conserved, 2-species conserved and species-specific RELA peaks to the transcription start sites (TSS) of genes in TNFα-stimulated (45-min, 10 ng/mL) HAECs, MAECs, and BAECs. **h,** Comparison of RELA peaks (Venn diagram) and ChIP-seq signals (genomic profiles) for RELA and H3K27ac (Reads per Million: RPM) centered on RELA peak summits between TNFα-stimulated HAECs and TeloHAECs (45-min TNFα, 10 ng/mL).

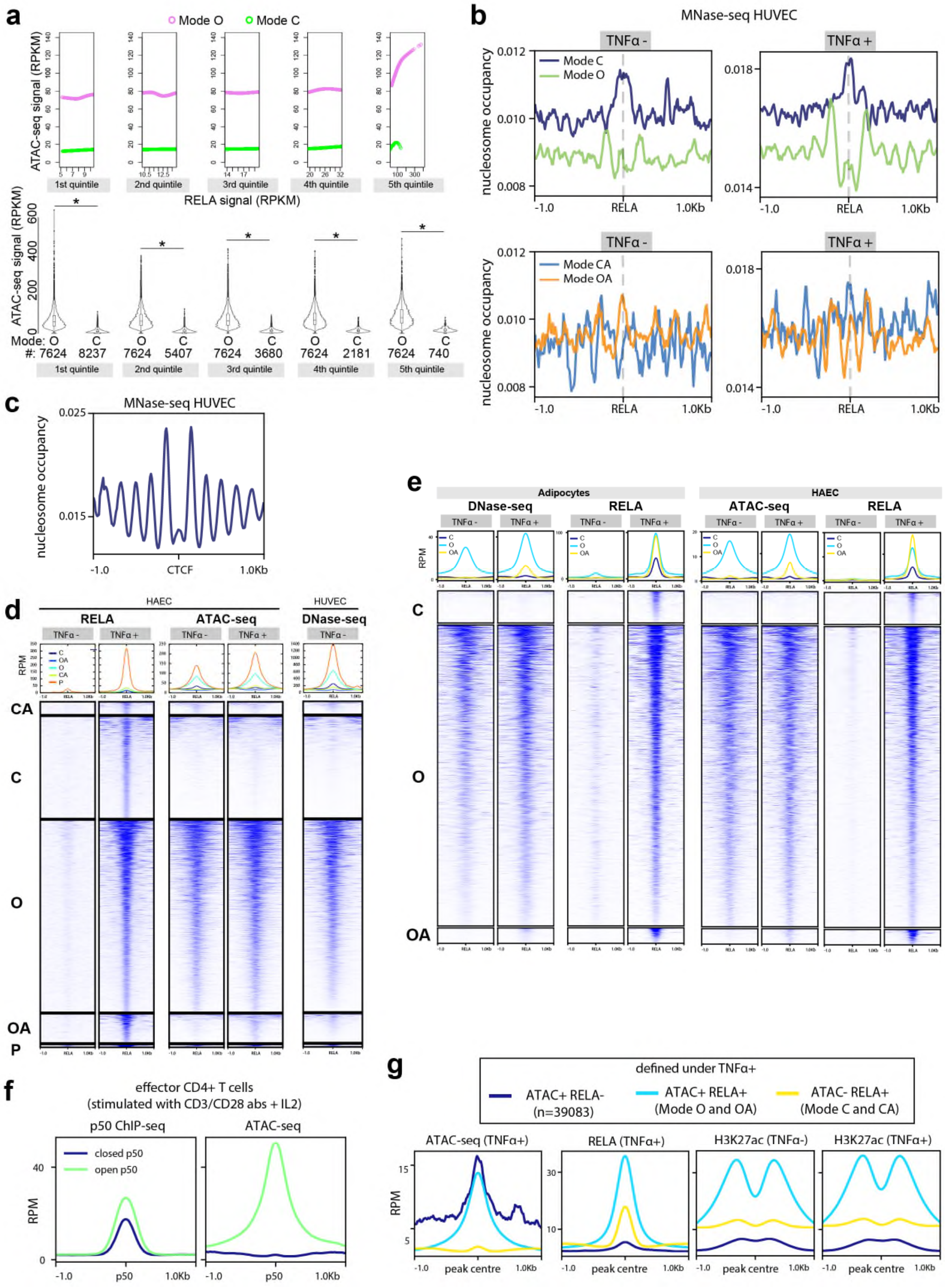

a
Mode O
Mode C
ATAC-seq signal (RPKM)
1st quintile
2nd quintile
3rd quintile
4th quintile
5th quintile
RELA signal (RPKM)
ATAC-seq signal (RPKM)
Mode: O C O C O C O C O C
#: 7624 8237 7624 5407 7624 3680 7624 2181 7624 740
b
MNase-seq HUVEC
TNFα -
TNFα +
Mode C
Mode O
Mode CA
Mode OA
nucleosome occupancy
0.012
0.010
0.008
0.018
0.014
-1.0
RELA
1.0Kb
c
MNase-seq HUVEC
nucleosome occupancy
0.025
0.015
-1.0
CTCF
1.0Kb
d
HAEC
HUVEC
RELA
ATAC-seq
DNase-seq
TNFα -
TNFα +
RPM
CA
C
O
OA
P
e
Adipocytes
HAEC
DNase-seq
RELA
ATAC-seq
RELA
TNFα -
TNFα +
RPM
C
O
OA
f
effector CD4+ T cells
(stimulated with CD3/CD28 abs + IL2)
p50 ChIP-seq
ATAC-seq
closed p50
open p50
RPM
40
0
-1.0
p50
1.0Kb
g
defined under TNFα+
ATAC+ RELA-
(n=39083)
ATAC+ RELA+
(Mode O and OA)
ATAC- RELA+
(Mode C and CA)
ATAC-seq (TNFα+)
RELA (TNFα+)
H3K27ac (TNFα-)
H3K27ac (TNFα+)
RPM
15
5
30
10
-1.0
peak centre
1.0Kb

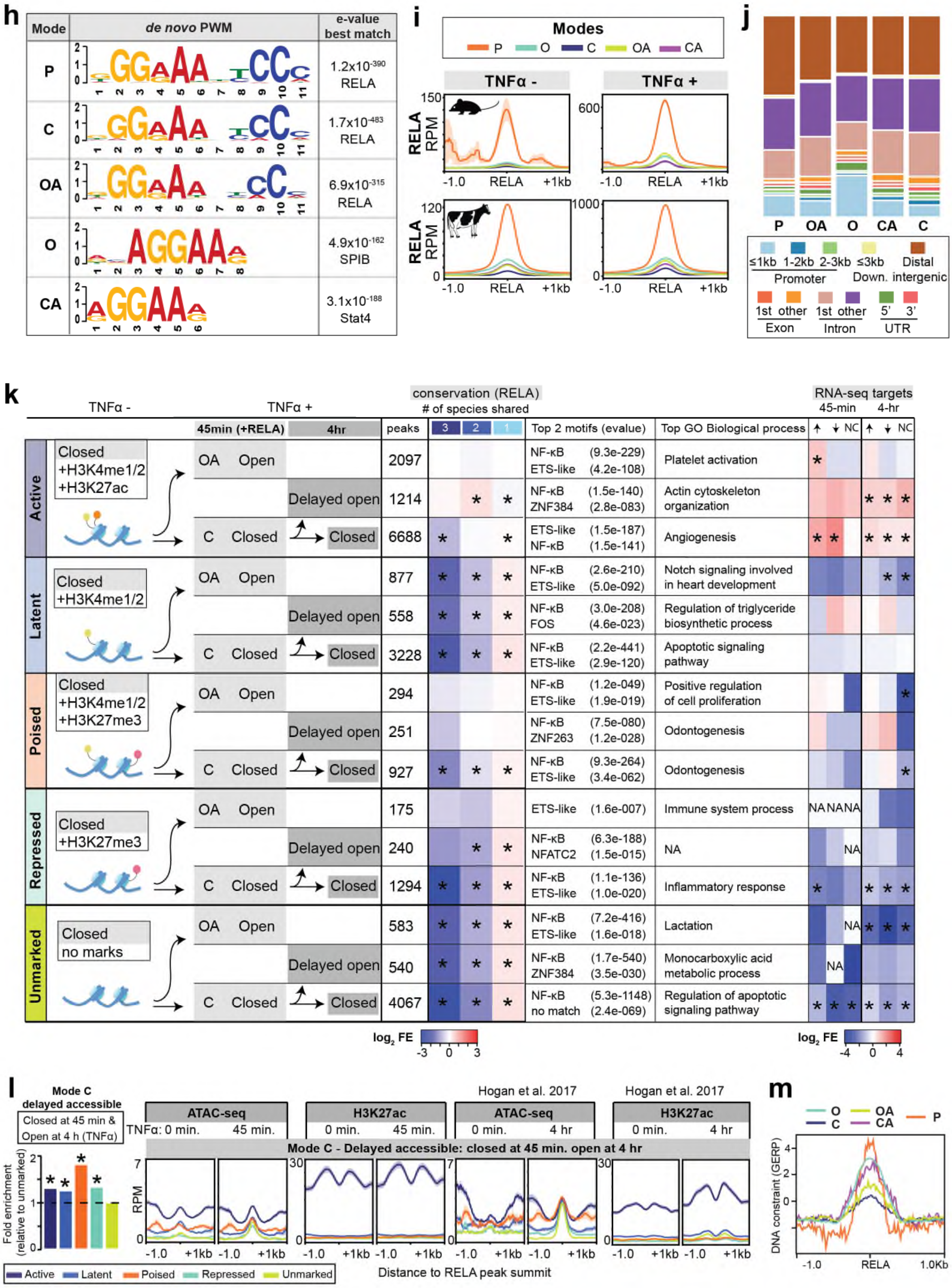
h
Mode
de novo PWM
e-value best match
P
1.2x10-390 RELA
C
1.7x10-483 RELA
OA
6.9x10-315 RELA
O
4.9x10-162 SPIB
CA
3.1x10-188 Stat4
i
Modes
P O C OA CA
TNFα -
TNFα +
RELA RPM
-1.0 RELA +1kb
j
P OA O CA C
≤1kb 1-2kb 2-3kb ≤3kb Distal
Promoter
Down. intergenic
1st other 1st other 5' 3'
Exon Intron UTR
k
TNFα -
TNFα +
conservation (RELA)
# of species shared
RNA-seq targets
45-min 4-hr
45min (+RELA) 4hr peaks 3 2 1 Top 2 motifs (evalue) Top GO Biological process NC NC
Active
Closed +H3K4me1/2 +H3K27ac
OA Open 2097 NF-κB (9.3e-229) ETS-like (4.2e-108) Platelet activation
Delayed open 1214 NF-κB (1.5e-140) ZNF384 (2.8e-083) Actin cytoskeleton organization
C Closed Closed 6688 ETS-like (1.5e-187) NF-κB (1.5e-141) Angiogenesis
Latent
Closed +H3K4me1/2
OA Open 877 NF-κB (2.6e-210) ETS-like (5.0e-092) Notch signaling involved in heart development
Delayed open 558 NF-κB (3.0e-208) FOS (4.6e-023) Regulation of triglyceride biosynthetic process
C Closed Closed 3228 NF-κB (2.2e-441) ETS-like (2.9e-120) Apoptotic signaling pathway
Poised
Closed +H3K4me1/2 +H3K27me3
OA Open 294 NF-κB (1.2e-049) ETS-like (1.9e-019) Positive regulation of cell proliferation
Delayed open 251 NF-κB (7.5e-080) ZNF263 (1.2e-028) Odontogenesis
C Closed Closed 927 NF-κB (9.3e-264) ETS-like (3.4e-062) Odontogenesis
Repressed
Closed +H3K27me3
OA Open 175 ETS-like (1.6e-007) Immune system process NA NA NA
Delayed open 240 NF-κB (6.3e-188) NFATC2 (1.5e-015) NA NA
C Closed Closed 1294 NF-κB (1.1e-136) ETS-like (1.0e-020) Inflammatory response
Unmarked
Closed no marks
OA Open 583 NF-κB (7.2e-416) ETS-like (1.6e-018) Lactation NA
Delayed open 540 NF-κB (1.7e-540) ZNF384 (3.5e-030) Monocarboxylic acid metabolic process NA
C Closed Closed 4067 NF-κB (5.3e-1148) no match (2.4e-069) Regulation of apoptotic signaling pathway
log2 FE -3 0 3
log2 FE -4 0 4
l
Mode C delayed accessible
Closed at 45 min & Open at 4 h (TNFα)
Fold enrichment (relative to unmarked)
Active Latent Poised Repressed Unmarked
Hogan et al. 2017
ATAC-seq H3K27ac ATAC-seq H3K27ac
TNFα: 0 min. 45 min. 0 min. 45 min. 0 min. 4 hr 0 min. 4 hr
Mode C - Delayed accessible: closed at 45 min. open at 4 hr
RPM
-1.0 +1kb
Distance to RELA peak summit
m
O OA C CA P
DNA constraint (GERP)
-1.0 RELA 1.0Kb

Supplementary Figure 2. Epigenomic properties of RELA-bound regions
a, Comparisons between ATAC-seq and RELA ChIP-seq signals (HAEC TNFα+ data) at Mode O and Mode C peaks. A cubic smoothing spline function smooth.spline was used in R to plot ATAC-seq data at each quantile of RELA ChIP-seq data (r=0.140 for Mode O and r=0.097 for Mode C, Pearson's product-moment correlation, $p<2.2 \times 10^{-16}$). Violin plots show differences in ATAC-seq signals between Mode O and C peaks within each quantile of RELA signal data (p value: * $p<1.0 \times 10^{-15}$, two-sided Welch Two Sample t-test and Bonferroni correction for multiple testing). Boxplot boundaries indicate 25th and 75th percentiles, the centre line indicates the median, whiskers represent the minimum and maximum of the data and small circles show outliers. **b,** MNase-seq data from HUVECs (Diermeier et al., 2014) was used to generate nucleosome occupancy profiles around RELA peak summits for each mode before and after TNFα stimulation. **c,** CTCF peaks (ENCODE HUVEC data from Pope et al., 2014) were used as a control to assess nucleosome profiles in the HUVEC MNase-seq data. **d,** Heatmaps showing how signals from HUVEC DNase-seq data (Neph et al., 2012) recapitulate HAEC ATAC-seq data at closed and open RELA binding modes. **e,** Heatmaps showing RELA binding modes identified using adipocyte DNase-seq and adipocyte RELA data (Schmidt et al., 2015) that are common with RELA binding modes identified using HAEC ATAC-seq and HAEC RELA data. There are 11,303 Mode C regions in Adipocytes of which 1100 are also Mode C in HAECs, 54,603 Mode O regions in Adipocytes of which 10,343 are also Mode O in HAECs, and 11,884 Mode OA regions in Adipocytes of which 527 are also Mode OA in HAECs. **f,** NF-κB binding modes at open and closed chromatin identified using peaks called from ChIP-seq of the p50 of NF-κB and ATAC-seq data obtained from activated CD4+ T cells sorted from two donors (Calderon et al., 2019). **g,** HAEC ATAC-seq, RELA, and H3K27ac signals compared between ATAC-seq+ RELA- peaks, ATAC-seq+ RELA+ and ATAC-seq- RELA- peaks. **h,** Position Weight Matrices (PWMs) of the top scoring *de novo* motifs (MEME-ChIP) for each RELA binding mode and their corresponding e-values. **i,** Profile plots showing RELA ChIP-seq signals in Reads per Million (RPM), with mapped reads centered on RELA peaks of different RELA binding modes in mouse aortic ECs (MAECs, top) and bovine aortic ECs (BAECs, bottom). The plot lines indicate mean RPM ± SEM (p value: * <0.05, two-sided Welch Two Sample t-test and Bonferroni correction for multiple testing). The modes have been defined as the following: Mode O (i.e. Open): RELA binding to accessible chromatin after TNFα stimulation, Mode C (i.e. Closed): RELA binding to inaccessible chromatin after TNFα stimulation, Mode OA (i.e. Open After): RELA binding to chromatin that becomes accessible after TNFα stimulation, Mode CA (i.e. Closed After): RELA binding to chromatin that becomes inaccessible after TNFα stimulation, Mode P (i.e. Pre-bound): RELA binding to accessible chromatin prior to TNFα stimulation. **j,** Stacked bar charts showing genomic feature annotations for RELA binding modes in HAECs (UCSC hg19 knownGene database). **k,** Table showing sub-classification of Mode C (i.e. Closed) and Mode OA (i.e. Open After) regions into early-accessible chromatin (45-min TNFα ATAC-seq peaks, our data), delayed-accessible chromatin (closed at 45-min and open 4-hr post-TNFα, based on ATAC-seq peaks generated using published raw data from Hogan et al., 2017), and constitutively closed chromatin subtypes using “active” (H3K4me2 or H3K4me1 + H3K27ac peaks), “latent” (H3K4me2 or H3K4me1 only peaks), “poised” (H3K4me2 or H3K4me1 + H3K27me3 peaks), “repressed” (H3K27me3 only peaks), and “unmarked” (no marks) chromatin states of unstimulated HAEC. Fold enrichments of 3-species conserved, 2-species conserved, and human-specific RELA peaks are shown for each subtype (p value: * $<5.5 \times 10^{-03}$, Chi-squared test with Yates's correction for continuity of independence and Bonferroni correction for multiple testing). The top scoring *de novo* motif

(MEME-ChIP e-values and matching TF names, Supplementary Table 4) and the top scoring GO functions (GREAT FDR q-values, Biological Process Ontology, Supplementary Table 3) are shown for each subtype. The fold enrichments of the RELA binding subtypes within -/+ 10 kb of the TSS of 45-min TNFα (total RNA-seq data, this study) and 4-hr TNFα (from published mRNA-seq data: Hogan et al., 2017) up-regulated (↑), down-regulated (↓), and constitutively expressed (NC: no change) HAEC genes are shown (right-hand side) (p value: * <0.05, two-sided Fisher's exact test, Bonferroni correction). **l,** Bar chart (left) showing fold enrichments for delayed-accessible Mode C peaks within chromatin marked with histone modifications as shown in panel k (p value: * $< 2.5 \times 10^{-04}$, Chi-squared test with Yates's correction for continuity of independence, Bonferroni correction). Profile plots (right) showing ATAC-seq and H3K27ac ChIP-seq signals in RPM, with mapped reads centered on RELA peaks of delayed-accessible Mode C subtypes classified in panel k. The plot lines indicate mean RPM ± SEM. NA=not available (peak is not present). **m,** DNA constraint (GERP score) of human RELA binding modes using hg19 GERP++ track data at UCSC. Source data are provided as a Source Data file for Supplementary Fig. 2k, l.

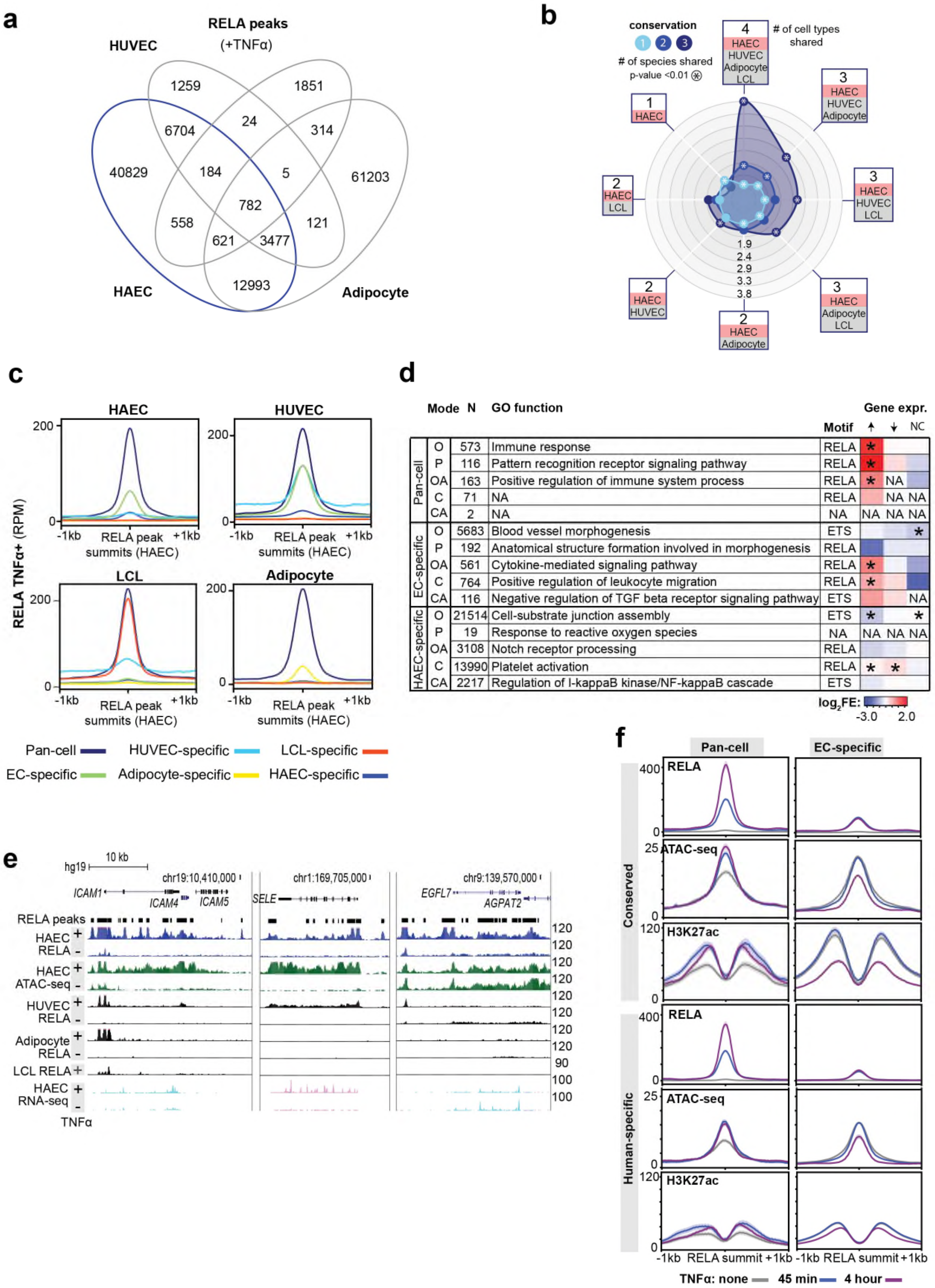
a
RELA peaks (+TNFα)
HUVEC
HAEC
Adipocyte
1259
1851
6704
24
314
40829
184
5
61203
782
558
121
621
3477
12993
b
conservation
1 2 3
# of species shared
p-value <0.01
# of cell types shared
4 HAEC HUVEC Adipocyte LCL
3 HAEC HUVEC Adipocyte
3 HAEC HUVEC LCL
3 HAEC Adipocyte LCL
2 HAEC Adipocyte
2 HAEC HUVEC
2 HAEC LCL
1 HAEC
1.9
2.4
2.9
3.3
3.8
c
RELA TNFα+ (RPM)
HAEC
HUVEC
LCL
Adipocyte
200
0
-1kb RELA peak summits (HAEC) +1kb
Pan-cell
HUVEC-specific
LCL-specific
EC-specific
Adipocyte-specific
HAEC-specific
d
Mode N GO function
Motif
Gene expr.
↑ ↓ NC
Pan-cell
O 573 Immune response RELA *
P 116 Pattern recognition receptor signaling pathway RELA *
OA 163 Positive regulation of immune system process RELA * NA
C 71 NA RELA NA NA
CA 2 NA NA NA NA NA
EC-specific
O 5683 Blood vessel morphogenesis ETS *
P 192 Anatomical structure formation involved in morphogenesis RELA
OA 561 Cytokine-mediated signaling pathway RELA *
C 764 Positive regulation of leukocyte migration RELA *
CA 116 Negative regulation of TGF beta receptor signaling pathway ETS NA
HAEC-specific
O 21514 Cell-substrate junction assembly ETS * *
P 19 Response to reactive oxygen species NA NA NA NA
OA 3108 Notch receptor processing RELA
C 13990 Platelet activation RELA * *
CA 2217 Regulation of I-kappaB kinase/NF-kappaB cascade ETS
log2FE: -3.0 2.0
e
hg19
10 kb
chr19:10,410,000
ICAM1
ICAM4
ICAM5
chr1:169,705,000
SELE
chr9:139,570,000
EGFL7
AGPAT2
RELA peaks
HAEC RELA + -
HAEC ATAC-seq + -
HUVEC RELA + -
Adipocyte RELA + -
LCL RELA +
HAEC RNA-seq + -
TNFα
120
120
120
120
120
120
120
120
90
100
100
f
Pan-cell
EC-specific
Conserved
Human-specific
RELA
ATAC-seq
H3K27ac
400
25
120
0
-1kb RELA summit +1kb
TNFα: none 45 min 4 hour

Supplementary Figure 3. Conserved RELA bound regions are often shared across tissues in proximity to genes that change expression after TNFα treatment

a, Venn diagram showing overlaps between RELA ChIP-seq peaks from TNFα-stimulated human aortic endothelial cells (HAECs) (45-min TNFα, 10 ng/mL, data from this study), human umbilical vein endothelial cells (HUVECs) (1-hr TNFα, 25 ng/mL, Brown et al., 2014), Simpson–Golabi–Behmel syndrome (SGBS) adipocytes (1-hr, 10 ng/mL, Schmidt et al., 2015), and lymphoblastoid cell lines (LCLs) (6-hr TNFα, 25 ng/mL, Kasowski et al., 2010). **b,** Radar plot indicating fold enrichments of the 3-species conserved, 2-species conserved, and human-specific RELA peaks within the 4-, 3-, and 2-cell-type-shared and HAEC-specific RELA peaks (1-cell type) (p value: * $<1.5 \times 10^{-03}$, Chi-squared test with Yates's correction for continuity of independence and Bonferroni correction for multiple testing). **c,** Profile plots comparing RELA ChIP-seq signal intensities in Reads per Million (RPM) between the pan-cell (4-cell-type-shared) and cell-type-specific RELA peaks of the TNFα-stimulated HAECs, HUVEC, Adipocytes, and LCLs. The plot lines represent mean RPM ± SEM. **d,** Table showing sub-classification of pan-cell, EC-specific, and HAEC-specific RELA-bound regions into Modes. The number of RELA-bound regions (N), the top scoring *de novo* motif (MEME-ChIP) and enrichments near HAEC target genes are shown for each category. ↑: up-regulated, ↓: down-regulated, NC: no change (constitutively expressed). (p value: * <0.05, two-sided Fisher's exact test, Bonferroni correction). **e,** Genomic browser shots of EC-specific and HAEC-specific RELA peaks. Mode OA and C regions can be seen at *ICAM1* and *SELE* loci while Mode O regions are seen at *EGFL7* locus. The pan-cell Mode P region can also be observed at the 5' UTR of *ICAM1*. **f,** ChIP-seq, ATAC-seq and H3K27ac profile plots showing the TNFα-responsive dynamics (0-min, 45-min, and 4-hr TNFα) in RELA binding, chromatin accessibility, and H3K27ac levels centered on the summits of the pan-cell (4-cell-type-shared) and EC-specific RELA peaks that are human-specific and conserved (3-species). RELA and H3K27ac ChIP-seq signals and ATAC-seq signals from HAECs at 4-hr TNFα were generated using published raw data from (Hogan et al., 2017). NA=not available (peak is not present). Source data are provided as a Source Data file for Supplementary Fig. 3b, d.

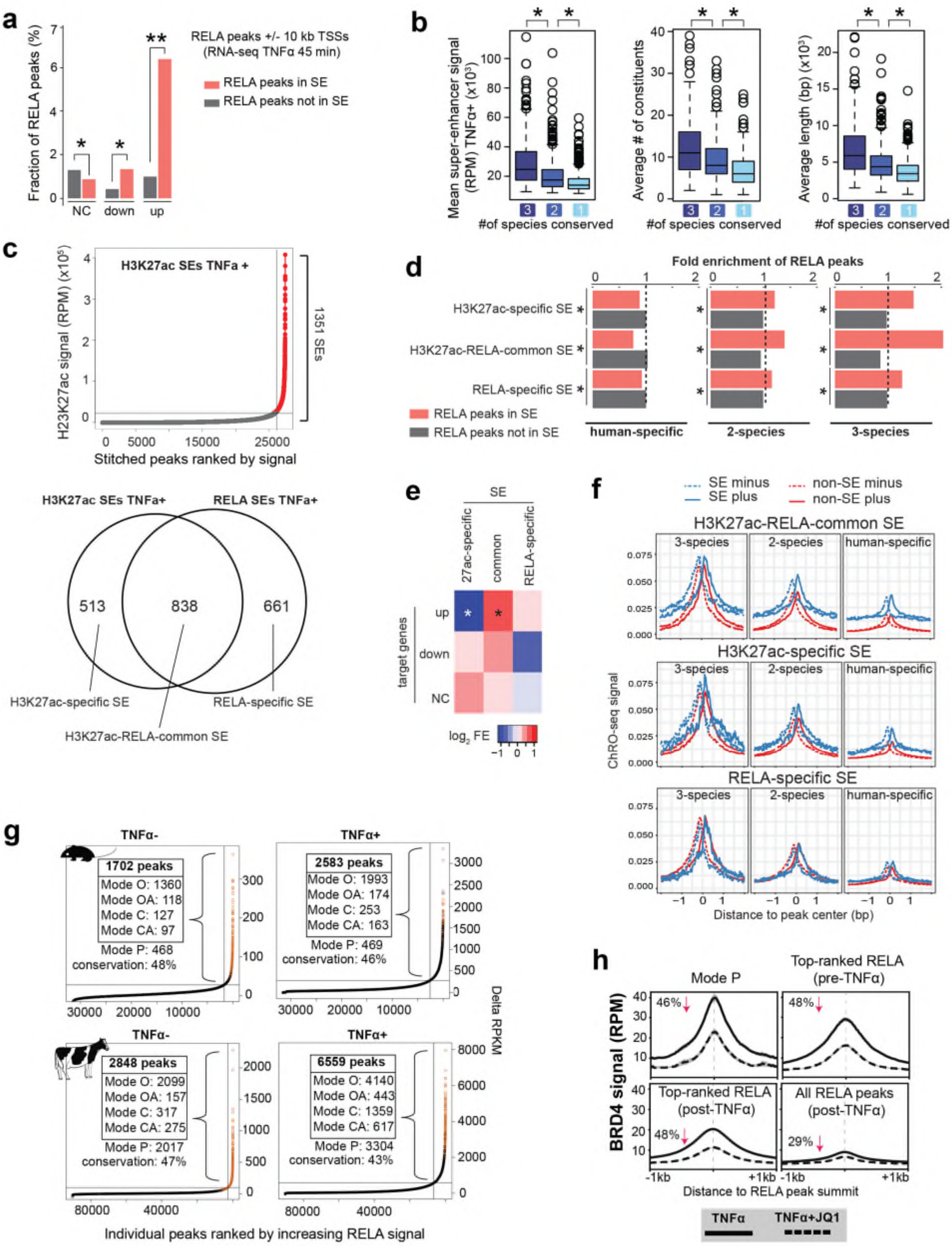

Supplementary Figure 4. Characterization of inflammatory super-enhancers and top-ranked RELA peaks.

a, Bar charts showing fractions of super-enhancer (SE) (pink) and non-SE (grey) RELA peaks that fall within +/- 10 kb of the TSS of TNFα up-regulated, down-regulated, and constitutively expressed (NC) genes in HAECs (p-value: * <5.0 x 10^{-4}, ** <1.0 x 10^{-251}, two-sided Fisher's exact test with Bonferroni correction for multiple testing). **b,** Boxplots comparing SE signal (Reads Per Million: RPM post-TNFα), constituent number and length (bp) between the 3-species conserved, the 2-species conserved, and the human-specific RELA SEs in HAECs (p-value: * <5.0 x 10^{-11}, two-sided Welch's t-test, Bonferroni correction for multiple testing). Boxplot boundaries indicate 25th and 75th percentiles, the centre line indicates the median, lower whiskers show minimum values while upper whiskers extend to the largest value that does not exceed 1.5 x IQR, and circles indicate outliers. **c,** H3K27ac SEs (red dots) in TNFα-stimulated (45-min) HAECs. The x-axis shows stitched RELA enhancers ranked by reads per million (RPM) signal (y-axis) (as derived from ROSE). The cut-offs defining the RELA SEs are indicated with vertical and horizontal lines. Venn diagram showing overlaps between H3K27ac and RELA SEs. **d,** Bar charts showing fold enrichments of the conserved RELA peaks within H3K27ac-specific, H3K27ac-RELA-common, and RELA-specific SEs in TNFα-stimulated HAECs. Fold enrichments for the 3-species conserved, 2-species conserved, human-specific RELA peaks are shown within SEs (pink) and non-SEs (grey) (p-value: * < 1.0 x 10^{-5}, Chi-squared test with Yates's correction for continuity of independence, Bonferroni correction for multiple testing). **e,** Heatmap indicating fold enrichments of SEs within +/- 10 kb of the TSS of TNFα target genes from HAEC RNA-seq analysis (p value: * <1.0 x 10^{-4}, two-sided Fisher's exact test with FDR correction for multiple testing). up=up-regulated, down=down-regulated, NC=no change (i.e. constitutively expressed). **f,** Profile plots showing ChRO-seq signals (TeloHAEC) at conserved and human-specific RELA peaks within and outside of H3K27ac-specific, H3K27ac-RELA-common, and RELA-specific SEs. **g,** Plots showing MAEC and BAEC RELA peaks ranked by normalized signal (RELA ChIP-seq Reads Per Kilobase per Million (RPKM) subtracted from cellular input RPKM) before and after TNFα stimulation. Mode P peaks are highlighted in orange. **h,** Changes in BRD4 occupancy in response to JQ1 treatment at Mode P and the top-ranked RELA binding regions in TNFα-treated HUVECs. BRD4 ChIP-seq signal was generated using published raw data from (Brown et al., 2014). The red arrows and percentages indicate changes in mean RPM signal at RELA peaks following the JQ1 treatment. The plot lines indicate mean RPM ± SEM. Source data are provided as a Source Data file for Supplementary Fig. 4a, b, d, e.

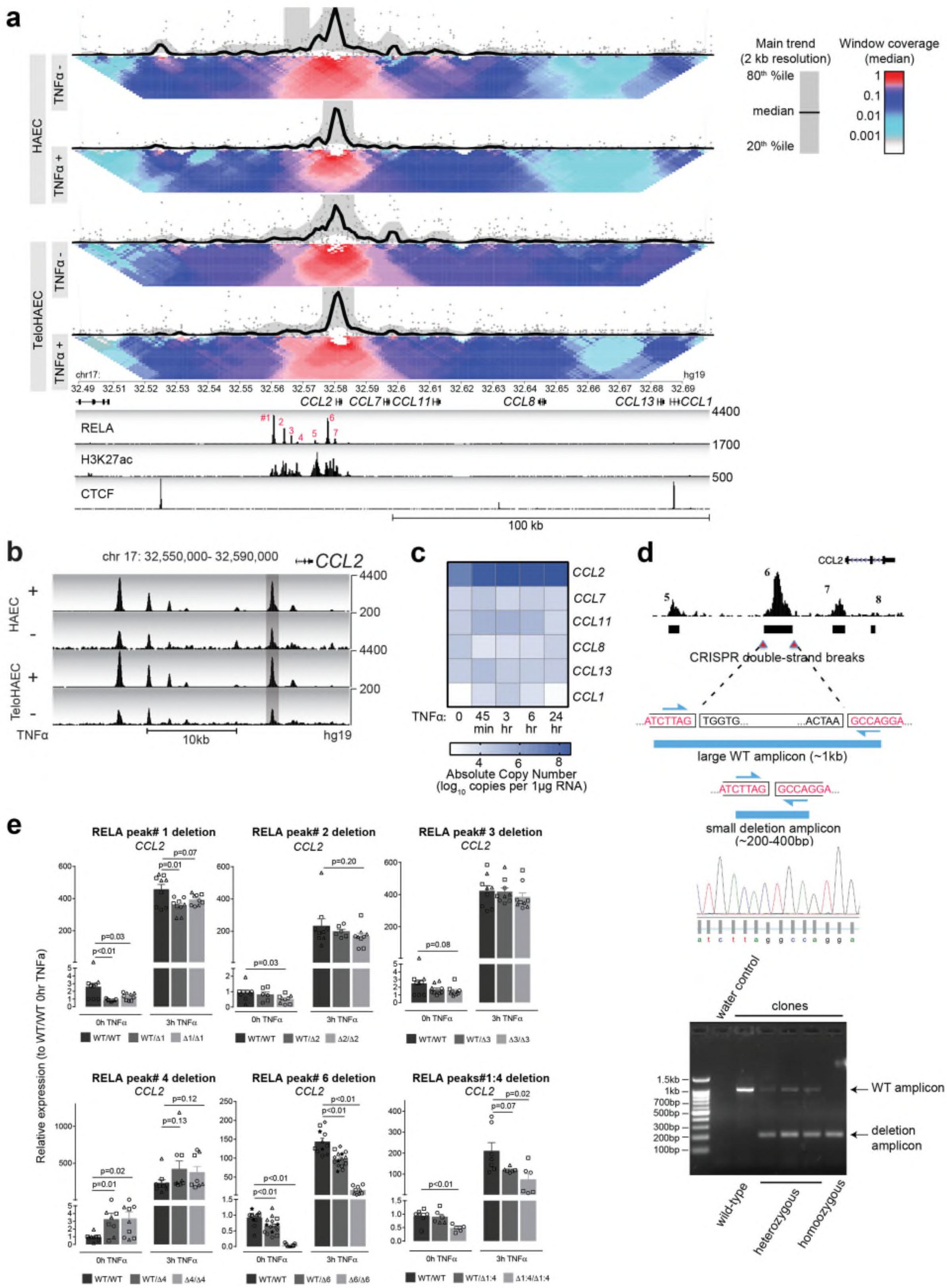
a
HAEC
TNFα -
TNFα +
TeloHAEC
TNFα -
TNFα +
Main trend (2 kb resolution)
80th %ile
median
20th %ile
Window coverage (median)
1
0.1
0.01
0.001
chr17:
hg19
CCL2 CCL7 CCL11 CCL8 CCL13 CCL1
RELA
H3K27ac
CTCF
4400
1700
500
100 kb
b
chr 17: 32,550,000- 32,590,000
CCL2
HAEC
TeloHAEC
TNFα
10kb
hg19
c
CCL2
CCL7
CCL11
CCL8
CCL13
CCL1
TNFα: 0 45 min 3 hr 6 hr 24 hr
Absolute Copy Number (log10 copies per 1μg RNA)
d
CRISPR double-strand breaks
large WT amplicon (~1kb)
small deletion amplicon (~200-400bp)
water control
clones
WT amplicon
deletion amplicon
wild-type
heterozygous
homoozygous
e
RELA peak# 1 deletion
RELA peak# 2 deletion
RELA peak# 3 deletion
RELA peak# 4 deletion
RELA peak# 6 deletion
RELA peaks#1:4 deletion
Relative expression (to WT/WT 0hr TNFα)
0h TNFα
3h TNFα

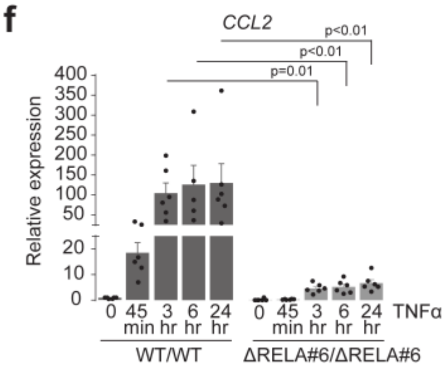

Supplementary Figure 5. CRISPR/Cas9-mediated genomic deletions in TeloHAECs reveal a strong effect of conserved RELA pre-bound regions on gene expression.

a, 4C-seq contact heatmaps of the *CCL2* promoter in HAECs and TeloHAECs before and after TNFα stimulation (top). Black lines show a median contact frequency over 2kb windows. Grey shaded area indicates 20th-80th percentiles. Colours indicate enrichments relative to the maximum attainable median value in a 12-kb window as calculated for sliding windows (2–50 kb) of linearly increasing size. A genome browser snapshot of a corresponding CCL locus showing ChIP-seq signals (RPM) for CTCF, H3K27ac, and RELA in the TNFα-stimulated HAECs (bottom). **b,** Comparison of RELA peaks at the *CCL2* super-enhancer locus between HAECs and TeloHAECs before and after TNFα-stimulation. RELA peak #6 is highlighted in grey. **c,** Heatmap of absolute copy numbers ($\log_{10}$) of *CCL* transcripts (RT-qPCR, standard curve) in unstimulated and TNFα stimulated (45-min, 3-hr, 6-hr, and 24-hr) TeloHAECs. **d,** Diagram depicting CRISPR double-strand breaks around RELA peak #6 and the expected PCR amplicon sizes for wild-type and deletion TeloHAEC clones. Gel electrophoresis results show the PCR products for wild-type, heterozygous and homozygous deletion clones. Sanger sequencing data shows the deletion sequence resulting from non-homologous end joining (NHEJ). The experiment was repeated more than ten times independently with similar results. **e,** Bar charts comparing *CCL2* expression (RT-qPCR, ΔΔCT) between the wild-type TeloHAECs (WT/WT, white bars) and the clones that are heterozygous (WT/D) and homozygous (D/D) for the *CCL2* SE RELA #1, #2, #3, #4, #6 and #1:4 deletions. The data is plotted relative to unstimulated wild type TeloHAECs (p values were derived using one-way ANOVA with Bonferroni correction). Error bars represent SEM. **f,** Bar charts comparing the TNFα time-course (0-min, 45-min, 3-hr, 6-hr, and 24-hr) of *CCL2* expression (RT-qPCR, ΔΔCT) between the wild-type TeloHAECs (WT/WT, white bars) and the clones that are homozygous for the *CCL2* SE RELA #6 deletion (ΔRELA #6/ΔRELA #6). The data is plotted relative to unstimulated wild type TeloHAECs (p values were derived using one-way ANOVA with Bonferroni correction). Error bars represent SEM. Error bars represent SEM. Source data are provided as a Source Data file for Supplementary Fig. 5c, e, f.

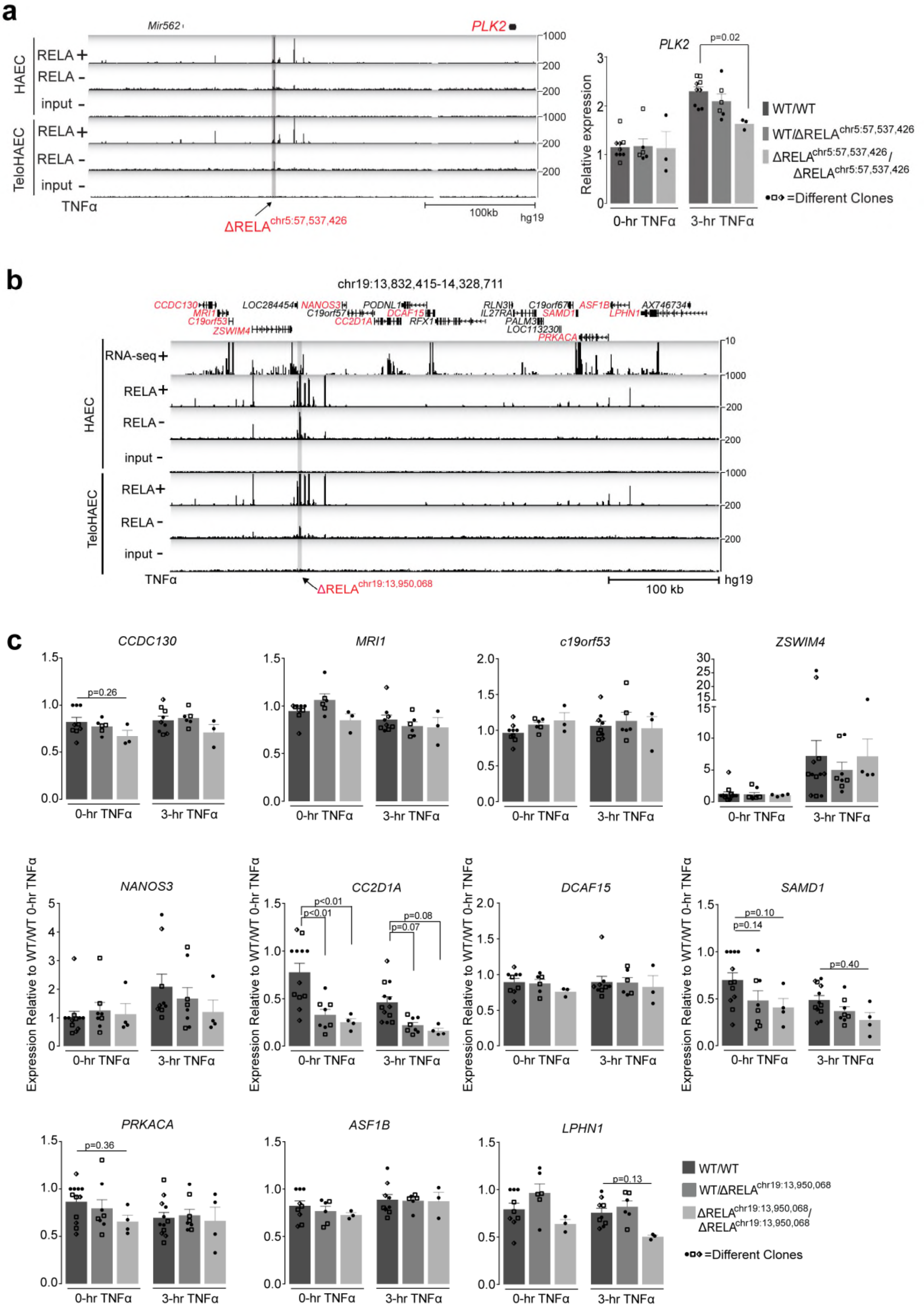
a
Mir562
PLK2
HAEC
TeloHAEC
RELA +
RELA -
input -
TNFα
ΔRELAchr5:57,537,426
100kb
hg19
PLK2
p=0.02
Relative expression
0-hr TNFα
3-hr TNFα
WT/WT
WT/ΔRELAchr5:57,537,426
ΔRELAchr5:57,537,426/ΔRELAchr5:57,537,426
=Different Clones
b
chr19:13,832,415-14,328,711
CCDC130
MRI1
C19orf53
ZSWIM4
LOC284454
NANOS3
C19orf57
CC2D1A
PODNL1
DCAF15
RFX1
RLN3
IL27RA
C19orf67
SAMD1
PALM3
LOC113230
PRKACA
ASF1B
LPHN1
AX747346
RNA-seq +
ΔRELAchr19:13,950,068
100 kb
c
CCDC130
MRI1
c19orf53
ZSWIM4
NANOS3
CC2D1A
DCAF15
SAMD1
PRKACA
ASF1B
LPHN1
Expression Relative to WT/WT 0-hr TNFα
p=0.26
p<0.01
p=0.08
p=0.07
p=0.10
p=0.14
p=0.40
p=0.36
p=0.13
WT/ΔRELAchr19:13,950,068
ΔRELAchr19:13,950,068/ΔRELAchr19:13,950,068

Supplementary Figure 6. Deletion of a conserved RELA pre-bound region affects expression of several genes within a locus

a, Genomic regions showing the super-enhancer (SE) harbouring a Mode P region near the TNFα-target gene *PLK2*. The CRISPR/Cas9-deleted Mode P region is highlighted in grey. Bar charts are showing gene expressions (RT-qPCR, ΔΔCT) before and after TNFα induction (3-hr, 10 ng/mL) in TeloHAEC clones that are heterozygous or homozygous for the deletion. Each shape represents data from one deletion colony (p values were derived using one-way ANOVA with Bonferroni correction). Error bars represent SEM. **b,** Super-enhancer (SE) with conserved Mode P region near TNFα-target gene *ZSWIM4*. The CRISPR/Cas9-deleted Mode P region is highlighted in grey. **c,** Bar charts showing relative gene expressions (RT-qPCR, ΔΔCT) before and after TNFα induction (3-hr, 10 ng/mL) in TeloHAEC clones that are heterozygous or homozygous for the deletion. Each shape represents data from one deletion colony (p values were derived using one-way ANOVA with Bonferroni correction). Source data are provided as a Source Data file for Supplementary Fig. 6a, c.

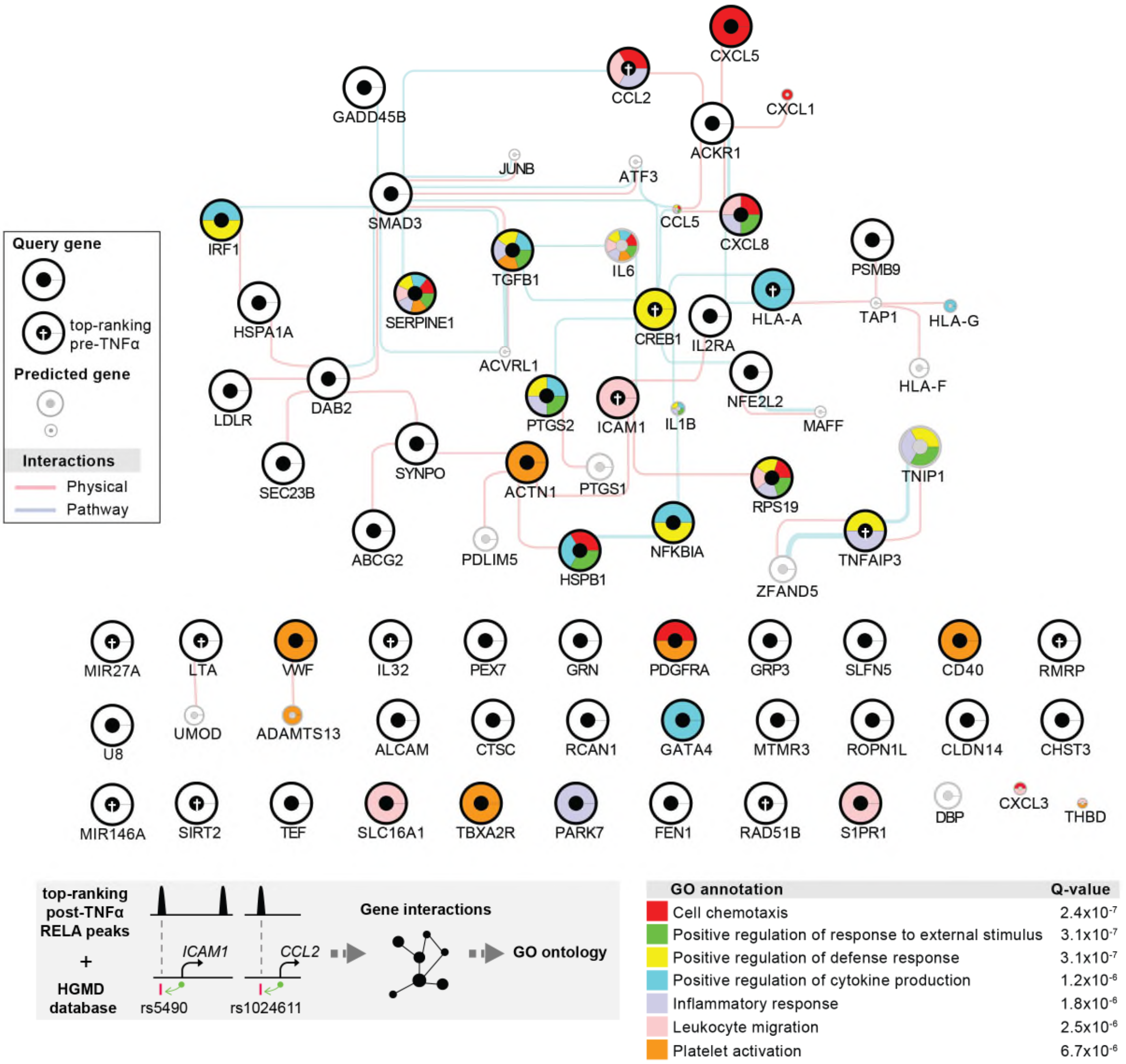

Supplementary Figure 7. Top-ranked RELA bound regions are associated with non-coding disease mutations in pro-inflammatory pathways and diseases.
Interaction network of genes (GeneMANIA) linked to the non-coding disease mutations (Human Gene Mutation Database) residing within the top-ranked post-TNFα RELA binding regions. The genes linked to mutations overlapping the top-ranked pre-TNFα RELA are labeled with a cross. The top non-redundant GO terms are shown for the genes (colors).
