# Supplementary material for: Conserved regulatory logic at accessible and inaccessible chromatin during the acute inflammatory response in mammals
Source: Nat Commun. 2021 Jan 25;12:567. doi: 10.1038/s41467-020-20765-1 (PMC7835376; doi:10.1038/s41467-020-20765-1)
Supplement: Supplementary file 3 — Description of Additional Supplementary Files [file 41467_2020_20765_MOESM3_ESM.pdf]

Description of Additional Supplementary Files

Supplementary Data 1: Summary of sequencing details and quality control for ChIP-seq, ATAC-seq, RNA-seq and ChRO-seq data generated.

Supplementary Data 2: Summary of conserved RELA binding and preserved RELA binding modes in human, mouse and bovine.

Supplementary Data 3: Complete lists of pathway enrichment analysis performed.

Supplementary Data 4: Complete results and position weight matrices for motif enrichments performed.

Supplementary Data 5: List of RT-qPCR primers and genotyping primers used.

Supplementary Data 6: List of Cas9 guides used.

Supplementary Data 7: Annotation of human RELA peak modes, conservation, and overlaps with HGMD mutations.

Supplementary Data 8: Detailed results for the RELI analysis comparing RELA binding to GWAS.
