# Supplementary material for: Conserved regulatory logic at accessible and inaccessible chromatin during the acute inflammatory response in mammals
Source: Nat Commun. 2021 Jan 25;12:567. doi: 10.1038/s41467-020-20765-1 (PMC7835376; doi:10.1038/s41467-020-20765-1)
Supplement: Supplementary file 5 — Supplementary Data 2 [file 41467_2020_20765_MOESM5_ESM.pdf]

Species	Modes	# of RELA peaks					Fold-enrichment of RELA peaks		
		Total	Conserved			Species-specific	Conserved	Species-specific	Corrected p-value*
			Total conserved	Mode preservation					
				Preserved	Not preserved				
HAEC	O	37464	13563	9641	3922	23901	1.27	0.89	0
	P	571	316	150	166	255	1.95	0.60	2.61×10^{-48}
	OA	5771	1413	149	1264	4358	0.83	1.07	1.25×10^{-17}
	C	20250	3885	2467	1418	16365	0.65	1.15	0
	CA	2663	677	103	574	1986	0.86	1.27	1.33×10^{-05}
MAEC	O	15606	6470	5369	1101	9136	1.25	0.87	1.69×10^{-213}
	P	559	279	110	169	280	1.51	0.75	1.28×10^{-16}
	OA	2562	758	103	655	1804	0.89	1.05	5.71×10^{-04}
	C	11678	2678	1396	1282	9000	0.69	1.15	1.40×10^{-188}
	CA	1756	549	87	462	1207	0.94	1.25	6.05×10^{-01}
BAEC	O	25241	9556	8510	1046	15685	1.78	0.78	0
	P	5314	2364	198	2166	2950	2.09	0.70	0
	OA	3870	800	111	689	3070	0.97	1.00	2.25
	C	53723	6822	2760	4062	46901	0.59	1.11	0
	CA	7736	2074	144	1930	5662	1.26	0.93	6.61×10^{-35}

Table S2. Summary of conserved RELA binding and preserved RELA binding modes.

* The p values are derived using Chi-squared test with Yates's correction for continuity of independence and Bonferroni correction for multiple testing
