# Supplementary material for: Conserved regulatory logic at accessible and inaccessible chromatin during the acute inflammatory response in mammals
Source: Nat Commun. 2021 Jan 25;12:567. doi: 10.1038/s41467-020-20765-1 (PMC7835376; doi:10.1038/s41467-020-20765-1)
Supplement: Supplementary file 9 — Supplementary Data 6 [file 41467_2020_20765_MOESM9_ESM.pdf]

Table S6. gRNA sequences

Deletion	5’ gRNA	3’ gRNA	Deletion Size
CCL2 RELA #1	GTATTTAAAGCACGTAACAC	TTCAGTCATTACTAGTCAGC	1100 bp
CCL2 RELA #2	CCTTGCTGGACCTAGGAACT	TTGACGTTTACCCCACACAA	600 bp
CCL2 RELA #3	TGCTAGATACTTGGGATACC	ATCTTCCATCTATGTGGCCC	670 bp
CCL2 RELA #4	CAAAGTCTTAATGTTTGGCC	TTTATGTGTCCAAACGAGTC	980 bp
CCL2 RELA #6	GGTAACTGAGGATTCTG	GTCTATAAGCCCATTGATTC	640 bp
CCL2 RELA #1:4	GTATTTAAAGCACGTAACAC	TTTATGTGTCCAAACGAGTC	8600 bp
CCL2 RELA #6 motif HR	TCTCGCTTGGGGACACACAA	CATTCTCTTCTACGGGATCT	X
PLK2 RELA	AACATGTCATCACGACAATT	GCCACAGAATGATAACTATG	630 bp
ZSWIM4 RELA	TATTATCCCTGTTTTGCAGA	TCACTAACGCTTTGCCTAAT	980 bp
