# Supplementary material for: Conserved regulatory logic at accessible and inaccessible chromatin during the acute inflammatory response in mammals
Source: Nat Commun. 2021 Jan 25;12:567. doi: 10.1038/s41467-020-20765-1 (PMC7835376; doi:10.1038/s41467-020-20765-1)
Supplement: Supplementary file 12 — Reporting Summary [file 41467_2020_20765_MOESM12_ESM.pdf]

nature research

Corresponding author(s): Michael D. Wilson, Jason E. Fish

Last updated by author(s): Nov 25, 2020

Reporting Summary

Nature Research wishes to improve the reproducibility of the work that we publish. This form provides structure for consistency and transparency in reporting. For further information on Nature Research policies, see Authors & Referees and the Editorial Policy Checklist .

Statistics

For all statistical analyses, confirm that the following items are present in the figure legend, table legend, main text, or Methods section.

n/a	Confirmed	
☐	☒	The exact sample size (*n*) for each experimental group/condition, given as a discrete number and unit of measurement
☐	☒	A statement on whether measurements were taken from distinct samples or whether the same sample was measured repeatedly
☐	☒	The statistical test(s) used AND whether they are one- or two-sided<br>*Only common tests should be described solely by name; describe more complex techniques in the Methods section.*
☐	☒	A description of all covariates tested
☐	☒	A description of any assumptions or corrections, such as tests of normality and adjustment for multiple comparisons
☐	☒	A full description of the statistical parameters including central tendency (e.g. means) or other basic estimates (e.g. regression coefficient) AND variation (e.g. standard deviation) or associated estimates of uncertainty (e.g. confidence intervals)
☐	☒	For null hypothesis testing, the test statistic (e.g. *F*, *t*, *r*) with confidence intervals, effect sizes, degrees of freedom and *P* value noted<br>*Give P values as exact values whenever suitable.*
☒	☐	For Bayesian analysis, information on the choice of priors and Markov chain Monte Carlo settings
☒	☐	For hierarchical and complex designs, identification of the appropriate level for tests and full reporting of outcomes
☐	☒	Estimates of effect sizes (e.g. Cohen's *d*, Pearson's *r*), indicating how they were calculated

Our web collection on statistics for biologists contains articles on many of the points above.

Software and code

Policy information about availability of computer code

Data collection	Illumina base-calling: bcl2fastq2 v2.20
Data analysis	Raw ChIP-seq and ATAC-seq data quality control: FastQC v0.11.8<br>Trimming adapter sequences: Trimmomatic v0.32<br>ChIP-seq and ATAC-seq Genome Alignment: bwa v0.7.8<br>Calling nucleosomes: NucleoATAC v0.3.4<br>Aligned ATACseq reads quality metrics: ataqv v1.0.0<br>RNA-seq quality control metrics: MultiQC v1.3<br>RNA-seq Genome Alignment: STAR v2.5.1b<br>Read count for RNA-seq: featureCounts v1.5.0<br>Peak Calling: MACS2 v2.1.1<br>4C-seq Analysis: 4Cseqpipe v0.7<br>Differential Enrichment Analysis: edgeR v3.18.1<br>Comparative analysis: 13-way eutherian mammals Enredo-Pecan-Ortheus (EPO) multiple sequence alignment (MSA) available in the Ensembl Compara multi-species database (Ensembl 70)<br>Peak overlaps: bedtools v2.23.0<br>Peak overlap visualization: UpSetR v1.4.0<br>Gene associations: GREAT v3.0 API<br>Gene interactions and networks: GeneMANIA prediction server v3.5.1 and Cytoscape v3.6.1<br>ChIP-seq and ATAC-seq heatmaps and profiles: deepTools2 v3.0.0<br>Scanning for RELA motifs: RSAT matrix-scan (http://rsat-tagc.univ-mrs.fr/rsat/matrix-scan-quick_form.cgi) (v 1.214)<br>Bean plots: beanplot v1.2<br>De novo motif discovery: MEME-ChIP (http://meme-suite.org/tools/meme-chip) in MEME Suite v5.1.0

ChRO-seq data analysis: proseq2.0 (https://github.com/Danko-Lab/proseq2.0)
Genomic annotations: ChIPseeker v1.12.1
ChIP-seq replicate validation: IDR (https://sites.google.com/site/anshulkundaje/projects/idr)
Super-enhancer calling: ROSE_main v0.1
MIT CRISPR design tool (http://crispr.mit.edu/) Software no longer available. Guide sequences used are provided.
4C primer designer tool: https://mnlab.uchicago.edu/4Cpd/ (Jan 25,2018 version)

For manuscripts utilizing custom algorithms or software that are central to the research but not yet described in published literature, software must be made available to editors/reviewers. We strongly encourage code deposition in a community repository (e.g. GitHub). See the Nature Research guidelines for submitting code & software for further information.

Data

Policy information about availability of data

All manuscripts must include a data availability statement. This statement should provide the following information, where applicable:
- Accession codes, unique identifiers, or web links for publicly available datasets
- A list of figures that have associated raw data
- A description of any restrictions on data availability

All ChIP-seq, ATAC-seq, RNA-seq, ChRO-seq, and 4C-seq data generated in this study have been submitted to the ArrayExpress database (http://www.ebi.ac.uk/arrayexpress) under accession numbers E-MTAB-7889, E-MTAB-7878, E-MTAB-7896, E-MTAB-8272, and E-MTAB-9425 respectively. Further information and requests should be directed to Michael D. Wilson (michael.wilson@sickkids.ca).

For the inter-tissue and epigenetic comparative analyses, raw ChIP-seq data from HUVECs, LCLs, HeLa cells, Adipocytes, and HAECs were downloaded from GEO database (GSE54000, GSE19486, GSE24518, GSE64233, GSE89970, respectively) and processed as described in methods. The raw ATAC-seq data from the 4-hr TNFα stimulated HAECs were downloaded from GEO database (GSE89970) and processed as described in methods. To identify the significant TNFα responsive genes at 4-hr TNFα induction of HAECs from previously published gene expression data, we downloaded the publicly available raw RNA-seq data from GEO database (GSE89970) and processed only the exonic reads as described in methods.

The publicly available processed data for HUVEC DNase-seq, HUVEC MNase-seq, Adipocyte DNase-seq and raw data for T-cell p50 ChIP-seq and T-cell ATAC-seq were downloaded from GEO database (https://www.ncbi.nlm.nih.gov/geo/) under accession numbers GSE26328, GSE53343, GSE64233, GSE126505, and GSE118189, respectively.

ChRO-seq data from TeloHAEC cell line:
link: http://www.ebi.ac.uk/arrayexpress/experiments/E-MTAB-9425

GWAS analysis with RELI: https://zenodo.org/record/4266978#.X77dphNKjUI

Source code for peak enrichment, differential analysis, and motif density analysis are available on Wilson lab GitHub repository (https://github.com/wilsonlabgroup/comparativeRELA) and (DOI: 10.5281/zenodo.4281310).

Field-specific reporting

Please select the one below that is the best fit for your research. If you are not sure, read the appropriate sections before making your selection.

☒ Life sciences ☐ Behavioural & social sciences ☐ Ecological, evolutionary & environmental sciences

For a reference copy of the document with all sections, see nature.com/documents/nr-reporting-summary-flat.pdf

Life sciences study design

All studies must disclose on these points even when the disclosure is negative.

Sample size	Two biological replicates for HAECs, TeloHAECs, MAECs, and BAECs were used for ChIP-seq, RNA-seq, ATAC-seq, and 4C-seq experiments. Three biological replicates of TeloHAECs (three different clonal populations from both homozygous and heterozygous CRISPR/Cas9 deletions) were used for RT-qPCR for CRISPR/Cas9 deletion and recombination experiments. Sample size was chosen based on established norms for ChIP-seq experiments that aim to annotate mammalian genomes (such as has been done by ENCODE).
Data exclusions	ATAC-seq data for BAEC TNFα sample replicate #1165 did not generate enough peaks and was excluded from analysis.
Replication	The reproducibility of RELA ChIP-seq peaks between the two biological replicates was assessed using the irreproducible discovery rate (IDR) statistic. The RELA peaks in the mouse and bovine cells were also recapitulated using an independent antibody (n=1 for each species).
Randomization	*Describe how samples/organisms/participants were allocated into experimental groups. If allocation was not random, describe how covariates were controlled OR if this is not relevant to your study, explain why.*
Blinding	Blinding was not relevant for our study. All species and treatments were labelled and analyses were done with these labels known to the investigator.

Reporting for specific materials, systems and methods

We require information from authors about some types of materials, experimental systems and methods used in many studies. Here, indicate whether each material, system or method listed is relevant to your study. If you are not sure if a list item applies to your research, read the appropriate section before selecting a response.

Materials & experimental systems

n/a	Involved in the study
☐	☒ Antibodies
☐	☒ Eukaryotic cell lines
☒	☐ Palaeontology
☒	☐ Animals and other organisms
☒	☐ Human research participants
☒	☐ Clinical data

Methods

n/a	Involved in the study
☐	☒ ChIP-seq
☐	☒ Flow cytometry
☒	☐ MRI-based neuroimaging

Antibodies

Antibodies used

rabbit anti-RELA polyclonal (Santa Cruz sc372 and Abcam ab7970); mouse anti-H3K27ac monoclonal (Millipore # 05-1334), rabbit anti-H3K4me2 polyclonal (Millipore # 07-030), mouse anti-H3K4me3 monoclonal (Millipore # 17-678), rabbit anti-H3K27me3 polyclonal (Millipore # 07-449), rabbit anti-H3K4me1 polyclonal (Abcam; ab8895), and rabbit anti-CTCF polyclonal (Millipore # 07-729), donkey anti-rabbit Cy3-conjugated secondary IgG antibody (AP182C, Sigma-Aldrich)

Validation

Millipore #05-1334: 5 citations for ChIP (citeab.com)
Millipore #07-030: 249 citations for ChIP (citeab.com)
Millipore #17-678: 7 citations for ChIP (citeab.com)
Abcam #ab7970: 42 citations for ChIP (citeab.com)
Abcam #ab8895: 318 citations for ChIP (citeab.com)
Santa Cruz #sc372: 58 citations for ChIP (citeab.com)
Millipore #07-449: 565 citations for ChIP (citeab.com)
Millipore #07-729: 111 citations for ChIP (citeab.com)
Sigma-Aldrich #AP182C: 50 citations for ChIP (citeab.com)

Eukaryotic cell lines

Policy information about cell lines

Cell line source(s)

TeloHAEC (purchased from ATCC #CRL-4052)

Authentication

Positive for CD31/PECAM-1 expression and capable of uptaking Low Density Lipoprotein (LDL); the cells also show effective inflammatory response upon TNFα treatment and increase proliferation upon VEGF stimulation. When co-cultured with fibroblasts, TeloHAEC cells can also form neoangiogenic tubular networks in vitro, which are responsive to VEGF stimulation and suramin inhibition. This is a diploid cell line of female origin with a consistent normal kayrotype at low and high passages

Mycoplasma contamination

Cell lines tested negative for mycoplasma by PCR

Commonly misidentified lines
(See ICLAC register)

No commonly misidentified lines were used in this study.

ChIP-seq

Data deposition

☒ Confirm that both raw and final processed data have been deposited in a public database such as GEO.

☒ Confirm that you have deposited or provided access to graph files (e.g. BED files) for the called peaks.

Data access links
May remain private before publication.

ChIP-seq
link: http://www.ebi.ac.uk/arrayexpress/experiments/E-MTAB-7889

4C-seq
link: http://www.ebi.ac.uk/arrayexpress/experiments/E-MTAB-8272

ATAC-seq
link: http://www.ebi.ac.uk/arrayexpress/experiments/E-MTAB-7878

RNA-seq
link: http://www.ebi.ac.uk/arrayexpress/experiments/E-MTAB-7896

ChRO-seq
link: http://www.ebi.ac.uk/arrayexpress/experiments/E-MTAB-9425

Files in database submission: fastq and bam

Genome browser session (e.g. UCSC):
Human data: https://genome.ucsc.edu/s/alizadaa/am
Cow data: https://genome.ucsc.edu/s/alizadaa/am.btau
Mouse data: https://genome.ucsc.edu/s/alizadaa/am.mm10

Methodology

Replicates: human (two 21 year old Caucasian males, lot# 2139 and 1487; a 15 year old male, lot# 2102; and a 60 year old male, lot# 2366; Cell Application cat# 304-05a), mouse (two biological replicates of C57BL/6 males pooled from multiple mice, Cell Biologics cat# C57-6052, lot# A092913T2MP and B092913T2MP), cow (two biological replicates, Cell Applications cat# B304-05, lot# 1165 and 1190), TeloHAECs (ATCC CRL-4052; two independently grown cell culture populations)

Sequencing depth: ChIP-seq: Illumina HiSeq2500 with 100-bp single-end run to obtain ~20-25 million single end reads per sample

Antibodies: rabbit anti-RELA polyclonal (sc372 and ab7970); mouse anti-H3K27ac monoclonal (Millipore # 05-1334), rabbit anti-H3K4me2 polyclonal (Millipore # 07-030), mouse anti-H3K4me3 monoclonal (Millipore # 17-678), rabbit anti-H3K27me3 polyclonal (Millipore # 07-449), rabbit anti-H3K4me1 polyclonal (ab8895), and rabbit anti-CTCF polyclonal (Millipore # 07-729)

Peak calling parameters: ChIP-seq peaks were called relative to the input (whole cell extract) data using MACS2 with false-discovery rate (FDR) cut-off $q \leq 0.01$. The broad option was used for calling histone peaks.

Data quality: ChIP-seq data was assessed with quality control metrics of the ENCODE consortium for PCR bottleneck coefficient (PBC), normalized strand coefficient (NSC), non-redundant fraction (NRF) and relative strand coefficient (RSC). The reproducibility of RELA ChIP-seq peaks between the biological replicates was assessed using the irreproducible discovery rate (IDR) statistic.

Software:
Raw ChIP-seq and ATAC-seq data quality control: FastQC v0.11.8
Trimming adapter sequences: Trimmomatic v0.32
Genome Alignment: bwa v0.7.8
Peak Calling: MACS2 v2.1.1

Flow Cytometry

Plots

Confirm that:

- [ ] The axis labels state the marker and fluorochrome used (e.g. CD4-FITC).
- [ ] The axis scales are clearly visible. Include numbers along axes only for bottom left plot of group (a 'group' is an analysis of identical markers).
- [ ] All plots are contour plots with outliers or pseudocolor plots.
- [ ] A numerical value for number of cells or percentage (with statistics) is provided.

Methodology

Sample preparation: *Describe the sample preparation, detailing the biological source of the cells and any tissue processing steps used.*

Instrument: *Identify the instrument used for data collection, specifying make and model number.*

Software: *Describe the software used to collect and analyze the flow cytometry data. For custom code that has been deposited into a community repository, provide accession details.*

Cell population abundance: *Describe the abundance of the relevant cell populations within post-sort fractions, providing details on the purity of the samples and how it was determined.*

Gating strategy	*Describe the gating strategy used for all relevant experiments, specifying the preliminary FSC/SSC gates of the starting cell population, indicating where boundaries between "positive" and "negative" staining cell populations are defined.*

☐ Tick this box to confirm that a figure exemplifying the gating strategy is provided in the Supplementary Information.
